# Prediction of preventive behaviors of cutaneous leishmaniasis in the endemic areas of northwest Iran: an application of BASNEF model

**DOI:** 10.1038/s41598-024-64600-9

**Published:** 2024-06-15

**Authors:** Eslam Moradi-Asl, Arman Latifi, Mahsa Rashtbari, Abbas Abbasi-Ghahramanloo, Sara Rahimi

**Affiliations:** 1 Arthropod-Borne Diseases Research Center, Ardabil University of Medical Sciences, Ardabil, Iran; 2 Department of Public Health, School of Health, Ardabil University of Medical Sciences, Ardabil, Iran; 3 Department of Public Health, Research Center for Evidence-Based Health Management, Maragheh University of Medical Sciences, Maragheh, Iran; 4 Medicinal Plants Research Center, Maragheh University of Medical Sciences, Maragheh, Iran

**Keywords:** Cutaneous leishmaniasis, Behavioral intention, Preventive behavior, BASNEF model, Iran, Diseases, Health care, Medical research

## Abstract

Cutaneous leishmaniasis (CL) is a disease transmitted by mosquitoes and is endemic in many regions of the world and Iran, and annually imposes a large burden on the health system. This study was conducted to identify the effective factors in the preventive behaviors of CL based on the BASNEF model in endemic areas in the northwest of Iran. This cross-sectional study was conducted in Bileh-Savar, ‘endemic areas of Ardabil Province, Iran’, from June 2022 to October 2022. 200 non-patients were included in the study by a multi-stage sampling method. A standard questionnaire based on the BASNEF model was applied for data collection. The data were analyzed using SPSS version 25. Means and standard deviations were calculated to describe the continuous variables, and multivariate linear regression analysis was used to determine the prediction of intention and behavior by the model structures. The BASNEF constructs predict 27% of behavioral intention changes. Among the constructs, attitude has a greater contribution in predicting changes (R^2^ = 0.27, *p* < 0.01). Also, the results showed that the BASNEF constructs predict 23% of behavior changes. Among the constructs, enabling factors have a greater contribution to predicting changes (R^2^ = 0.23, *p* < 0.01). This finding shows that behavior is more influenced by environmental factors, and educational interventions based on behavior change models, along with providing environmental conditions, can facilitate behavior change.

Cutaneous leishmaniasis (CL) is a disease caused by the Leishmania parasite, which is transmitted by female sandflies^1^. The World Health Organization (WHO) reported that this group of neglected diseases is present in 98 countries, with 12 million cases potentially at risk and 20,000–40,000 fatalities annually^2,3^. Iran is one of the six nations with the highest prevalence of leishmaniasis in the world and one of the EMRO nations with the highest prevalence^4^. In Iran, there are about 20,000 cases of CL recorded annually, but there are probably more than four or five times that many actual cases. In Iran, CL has been reported in both rural and urban forms. The rural variant is prevalent in the majority of the country’s 15 provinces’ rural areas. Various risk factors, including environmental modifications due to urbanization and natural disasters, a lack of competent vector and reservoir control strategies, population displacements, drug resistance, poor sanitation, and human behaviors, play essential roles in the expansion of the disease^5–9^. Previous studies indicate that the knowledge of the community about CL is low^10–12^. Improving the level of health, knowledge, and preventive skills for personal protection in endemic regions is one of the most important and necessary strategies of the CL control program. Additionally, numerous studies have highlighted the significance of community involvement and health education in the field of leishmaniasis vector management. Effective health education should comprehensively and completely examine the situation^13^. The BASNEF model is used to define the factors that influence people’s decision-making and to analyze behaviors and strategies for changing them^14,15^. BASNEF (Beliefs, Attitudes, Subjective Norms, Enabling Factors, and Behavior) model in the context of behavior modification^16^. This model can be applied to the context of CL as follows: beliefs about the causes of CL, such as the role of sandfly bites and environmental factors, the severity and consequences of the disease, and the effectiveness of preventive measures. Attitudes towards seeking medical care for suspected CL lesions, and adopting preventive behaviors. Subjective Norms; the perceived social pressure from family members, community leaders, and healthcare providers to seek timely treatment and adopt preventive measures, the influence of cultural beliefs and practices on the perceived social norms related to CL. Enabling Factors; access to healthcare services for diagnosis and treatment of CL, availability and affordability of preventive measures, such as insect repellents and bed nets. Behavior; seeking early medical attention for suspected CL lesions. By understanding the interplay of these model components, researchers and public health professionals can develop targeted interventions to address the complex behavioral factors that influence the prevention and control of cutaneous leishmaniasis in affected communities^15,17,18^. Based on our knowledge, few studies have been conducted in relation to the application of BASNEF model in identifying cognitive factors effective on preventive behaviors of CL. Therefore, this study was conducted to identify the effective factors in the preventive behaviors of CL based on the BASNEF model in endemic areas in the northwest of Iran.

## Methods

### Study design and sampling

This cross-sectional study was conducted in Bileh-Savar, the city located in Ardabil province, the northwest of Iran, and the border of the Republic of Azerbaijan, from June 2022 to October 2022. This district is endemic to leishmaniasis. The sample size was estimated to be 200 participants according to the previous studies and the Krejcie -Morgan standard table. Sampling was conducted in a multi-stage method. First, two health centers in the city were considered strata (there are only two health centers in the city); then, using a table of random numbers and based on the file number of the households in each center, 100 people were selected and invited to participate in the study.

### Inclusion and exclusion criteria

People living in the city, and having no previous history of leishmaniasis were included in the study. People with less than 6 months of residence in the city people who were unable to communicate effectively due to language barriers, and people who did not want to complete the questionnaires were excluded from the study.

### Data collection method

A three-part standard questionnaire was used as the data-gathering tool. Previous studies have approved the questionnaire’s validity and reliability^17^. Age, gender, education level, and other demographic data were covered in the first section. Five questions were assessed the knowledge about CL, like “CL transmitted by mosquitoes,” comprised the second section. True, false, and I don’t know where the three options were used to rate the items; accurate answers received a score of 1, while incorrect and I don’t know responses received a score of 0. The BASNEF model constructs were the subject of the third section’s questions.

Six statements, such as “If I practice the CL’s preventive behaviors, I will not get the disease,” were used to assess attitude. The total scores, which varied from 0 to 24, were the sum of the item scores.

Eight items, like “My family expects me to practice CL prevention behaviors,” were used to assess subjective norms. The total scores, which varied from 0 to 32, were the sum of the item scores.

Five items made up enabling factors, including “I don’t have enough money to buy insect repellent and insecticides.” The total scores, which varied from 0 to 20, were the sum of the item scores.

Eight items, such as “I intend to use a mosquito net when sleeping for the next two months,” assessed the behavioral intentions. The total scores ranged from 0 to 32 after the item scores were combined.

Behavior was measured by five items, like “to prevent mosquitoes from entering, I install a net on the door and windows of the house”.

A five-point Likert scale, ranging from 0 to 4, was used to assess the items that examined the BASNEF components. There were five options: completely agree, agree, undecided, disagree, and completely disagree. The behavior measurement scale was always, often, sometimes, rarely, and never.

### Statistical analysis

The data were analyzed using SPSS version 25. We first employed descriptive statistics to summarize the demographic characteristics of the participants as well as the scores on the main variables of interest. Additionally, we used means and standard deviations to describe the continuous variables. The normality of the distributions was assessed using histograms, Q-Q plots, and summary statistics to examine the data. The correlations between the independent and dependent variables were examined employing Pearson’s r. The multiple linear regression models were fitted using the enter method, with the dependent variables (intention and behavior) being the outcome of interest and the independent variables (knowledge, attitude, subjective norms, enabling factors, and intention) being the predictors. The assumptions of linearity, homoscedasticity, independence of errors, and multicollinearity were checked using diagnostic plots and tests. We reported the regression coefficients, standard errors, *p*-values, and 95% confidence intervals for each predictor, and also reported the R-squared and adjusted R-squared values to assess the overall fit of the model. The results are presented in tables, with the corresponding narratives in the text.

### Ethics approval and consent to participate

The Ardabil University of Medical Sciences Ethics Committee authorized the study protocol IR.ARUMS.REC.1398.646. Verbal informed consent was acquired. The research participants did not face any financial burdens as a result of their participation. The goal of the study was fully disclosed to the respondents, and they received assurances regarding the privacy of their personal information. Additionally, participants could leave the research at any time. The whole research was performed under the principles of the Declaration of Helsinki.

## Results

A total of 200 people completed the questionnaires. The mean and standard deviation of the age of the participants in the study were 35.94 ± 9.05 (19–62 years). The majority of the participants had an undergraduate degree (59.5%; *n* = 119). 67 percent (*n* = 134) had never heard anything about CL. Other characteristics are presented in Table 1.

**Table 1:** Demographic characteristics of the study participants (*n* = 200).

Characteristics		Frequency (No)	Percent (%)
Gender	Male	103	51.5
	Female	97	48.5
Educational level	Elementary	13	6.5
	Secondary	49	24.5
	Undergraduate	119	59.5
	Postgraduate	19	9.5
History of CL in family members	Yes	12	6
	No	188	94
Place of living	Downtown	73	36.5
	Outskirts of the city	67	33.5
	Village	60	30
Hearing about CL	Yes	66	33
	No	134	67
Livestock keeping	Yes	37	18.5
	No	163	81.5

The results showed that there is a positive and significant statistical correlation between attitude with knowledge (*r* = 0.32, *p* < 0.01), subjective norm (*r* = 0.38, *p* < 0.01), and, enabling factors (*r* = 0.29, *p* < 0.01). Also, a positive and significant statistical correlation was observed between enabling factors and subjective norms (*r* = 0.26, *p* < 0.01). The mean and standard deviation and, Pearson’s correlation coefficient between the constructs of the BASNEF model are shown in Table 2.

**Table 2:** Mean and standard deviation and, Pearson’s correlation coefficient between the constructs of the BASNEF model.

	K	A	S.B	E.F	I	B	Mean ± SD
K	1						2.21 ± 1.81
A	0.32**	1					19.40 ± 2.51
S.N	− 0.01	0.38**	1				24.96 ± 4.74
E.F	0.13	0.29**	0.26**	1			11.20 ± 4.29
I	0.29	0.47	0.30	0.03	1		23.62 ± 4.31
B	− 0.02	0.18	.34	0.44	0.15	1	12.09 ± 4.82

**Correlation is significant at the 0.01 level (2-tailed).

K (Knowledge), A (Attitude), S.B (Subjective Norms), E.F (Enabling Factors), I (Intention), and B (Behavior).

The results of multiple linear regression showed that the BASNEF constructs predict 27% of behavioral intention changes.

Given beta coefficients, the estimated change in behavioral intention is associated with a one-unit change in the independent variables (Knowledge, Attitude, Subjective Norms, and Enabling Factors), while holding all other variables constant. Compared to other constructs, attitude has a greater contribution in predicting changes (sdz ꞵ Coeff = 0.36, *R*^2^ = 0.27, *p* < 0.01) (Table 3). Also, the results of multiple linear regression showed that the BASNEF constructs predict 23% of behavior changes. Compared to other constructs, enabling factors have a greater contribution to predicting changes (sdz ꞵ Coeff = 0.40, R^2^ = 0.23, *p* < 0.01). (Table 4).

**Table 3:** The results of linear regression of BASNEF model constructs in predicting intention.

	Un-sdz ꞵ Coeff^a^	sdz ꞵ Coeff^b^	Adjusted R^2^	sig	95% CI
Constant	7.50		0.27	0.00	3.22, 11.78
K	0.45	0.19		0.00	0.14, 0.76
A	0.62	0.36		0.00	0.37, 0.85
S.N	0.18	0.20		0.00	0.06, 0.30
E.F	− 0.13	− 0.13		0.04	− 0.25, 0.00

^a^Unstandardized Beta Coefficients, b. Standardized Beta Coefficients.

K (Knowledge), A (Attitude), S.B (Subjective Norms), E.F (Enabling Factors).

**Table 4:** The results of linear regression of BASNEF model constructs in predicting behavior.

	Un-sdz ꞵ Coeff^a^	sdz ꞵ Coeff^b^	Adjusted R^2^	sig	95% CI
Constant	− 0.27		0.23	0.91	− 5.37, 4.84
K	− 0.28	− 0.10		0.13	− 0.64, 0.08
A	0.02	0.01		0.90	− 0.27, 0.31
S.N	0.20	0.20		0.00	0.05, 0.34
E.F	0.44	0.40		0.00	0.29, 0.58
I	0.12	0.10		0.16	− 0.04, 0.28

^a^Unstandardized Beta Coefficients, ^b^Standardized Beta Coefficients.

K (Knowledge), A (Attitude), S.B (Subjective Norms), E.F (Enabling Factors), I (Intention).

## Discussion

Behaviors are influenced by various factors, including a. personal or intrapersonal factors such as awareness, attitude, and belief of a person; b. interpersonal factors include communication and interaction of a person with others- significant others; c. institutional or organizational factors, d. social factors; and e. public laws and policies. It is very important to understand the influencing mechanisms of psychological factors such as subjective norms, attitudes, and other factors on the intention and consequences of behavior. Also, these psychological factors play a major role in determining the probability of acceptance or rejection of health behaviors^16^. Thus, the present study was conducted to identify the effective factors in the preventive behaviors of CL based on the BASNEF model in endemic areas in the northwest of Iran. The findings of this study showed that people’s knowledge about different dimensions of the CL is not desirable, and the majority of the participants had never heard anything about the CL. It is obvious that people’s lack of knowledge related to CL, which is one of the endemic diseases of the region, can act as a barrier to adopting preventive behaviors. The findings of other studies conducted in Iran also indicate the low level of awareness in the society about CL^14,19–23^. This is a serious warning because the low level of people’s knowledge can make other health and environmental interventions less effective. On the other hand, increasing the level of community knowledge in endemic areas is the first and most basic step for any appropriate behavior. The mean score of the attitude, subjective norm, and intentional behavior of the participants in the study was relatively appropriate, but the mean score of the enabling factors and preventive behavior of CL was low. The results of other studies are consistent with the present finding^15,22–24^. In the present study, knowledge was low, but a positive attitude towards CL preventive behaviors was observed. Much research in the field of health education is based on the knowledge, attitude, and performance (KAP) model. For some reason, this model cannot be a good tool for studying behavior in the health field. On the other hand, behavior is a complex phenomenon, and this framework does not seem to be sufficient for its explanation and prediction. In other words, a change in knowledge will not necessarily and simply lead to a modification of attitude and behavior. On the other hand, mediating factors are effective in the process of behavior change, which are not considered in this model^25^. The results showed that there is a positive and significant statistical correlation between attitude and knowledge, subjective norms, and enabling factors. Also, a positive and significant statistical correlation was observed between enabling factors and subjective norms. The result of multivariate regression analysis showed that 27 percent of changes in intention are explained by knowledge, attitude, subjective norms, and, enabling factors. Among these, attitude and subjective norms, respectively, had the most, and, enabling factors had the least effect on intention. This finding is consistent with previous studies^14,22,23^. Also, the result of multivariate regression analysis showed that 23 percent of changes in CL preventive behavior are explained by enabling factors (Standardized Beta Coefficients = 0.4, *p* < 0.00) and, subjective norms (Standardized Beta Coefficients = 0.2, *p* < 0.00). However, knowledge, attitude, and intention had no statistically significant role in predicting behavior. According to the previous study, 32% of behavior changes were predicted by the constructs of the BASNEF model, and the enabling factors had the highest effect, which is consistent with the results of the present study^20^. Also, the power of predicting different behaviors based on the BASNEF model has been reported in different studies^22,26,27^. Here, the important role of enabling factors should be considered as the prerequisites for a behavior or environmental change that allow the motivation of a behavior or environmental change to be realized. These factors can directly affect the behavior or indirectly through an environmental factor. New skills to change behavior, money, services, and resources needed to realize behavioral and environmental consequences are among these factors^16,28,29^. It seems that it is necessary to provide facilities such as insecticides and mosquito nets, as well as interventions to increase people’s skills and increase people’s motivation to adopt CL prevention behaviors. Health centers play a crucial role in educating the local population about CL its transmission, symptoms, and prevention measures. Health workers provide information through various channels, such as community meetings, school-based programs, and the distribution of educational materials. By grounding their health education and awareness initiatives in these well-established theories and models, health centers in Iran and other countries can develop more targeted, culturally appropriate, and effective interventions to combat CL and improve public health outcomes.

The present study had some limitations in study design and analysis, including reverse causality, incidence-prevalence bias, and unmeasured confounding.

## Conclusion

Applying behavioral change theories and models to identify the factors influencing the change in health behaviors, such as CL prevention, is crucial. The BASNEF model is also considered one of these models. In general, the result of this research showed that attitude, subjective norms, knowledge, and enabling factors have the greatest role in predicting behavioral intention, respectively. Also, enabling factors and subjective norms significantly predict CL preventive behavior. Although only 23% of behavior changes are predicted by this model, it is suggested that future studies be conducted with extended models that also examine other factors affecting behavior. This finding shows that behavior is more influenced by environmental factors, and educational interventions based on behavior change models, along with providing environmental conditions, can facilitate behavior change. Health education and awareness initiatives must be guided by established theories and models to ensure their effectiveness in promoting behavioral changes and improving health outcomes.

## Data Availability

The datasets used and analyzed during the current study are available from the corresponding author upon reasonable request.

## Abbreviations

CL: Cutaneous leishmaniasis
BASNEF: Beliefs and attitude subjective norms enabling factors

## References

[1] Control of Neglected Tropical Diseases. World Health Organization.Geneva: WHO; 2020. http:// www. who. int/ negle cted_ diseases/ disea ses/ en/#. Accessed 14 Oct 2023.

[2] Alvar, J. *et al.* Boer Md, Team WLC: Leishmaniasis worldwide and global estimates of its incidence. *PLoS ONE* **7**(5), e35671 (2012). 22693548 10.1371/journal.pone.0035671 PMC3365071

[3] WHO: World Health Organization, Leishmaniasis. Fact Sheet, WHO, 2019. https://www.who.int/news-room/fact-sheets/detail/leishmaniasis. Accessed 14 Oct 2023.

[4] Ahmadi, N., Ghafarzadeh, M. & Jalaligalosang, A. E. An epidemiological study of cutaneous leishmaniasis with emphasis on incidence rate in Kashan, Isfahan province. *J. Ilam. Uni. Med. Sci.* **21**(2), 1–9 (2013).

[5] Postigo, J. A. R. Leishmaniasis in the world health organization eastern mediterranean region. *Int. J. Antimicrob. Agents* **36**, S62–S65 (2010). 20728317 10.1016/j.ijantimicag.2010.06.023

[6] Desjeux, P. Leishmaniasis: current situation and new perspectives. *Comp. Immunol. Microbiol. Infect. Dis.* **27**(5), 305–318 (2004). 15225981 10.1016/j.cimid.2004.03.004

[7] Askari, A. *et al.* A newly emerged focus of zoonotic cutaneous leishmaniasis in South-Western Iran. *Microb. Pathog.* **121**, 363–368 (2018). 29709689 10.1016/j.micpath.2018.04.053

[8] Razavinasab, S. Z. *et al.* Expansion of urban cutaneous leishmaniasis into rural areas of southeastern Iran: Clinical, epidemiological and phylogenetic profiles explored using 7SL high resolution melting-PCR analysis. *Transbound. Emerg. Dis.* **66**(4), 1602–1610 (2019). 30912874 10.1111/tbed.13186

[9] Bamorovat, M. *et al.* Risk factors for anthroponotic cutaneous leishmaniasis in unresponsive and responsive patients in a major focus, southeast of Iran. *PLoS ONE* **13**(2), e0192236 (2018). 29415078 10.1371/journal.pone.0192236 PMC5802920

[10] Rakhshani, T., Kashfi, M., Ebrahimi, M. R., Taravatmanesh, S. & Rasheki, M. Knowledge, attitude and practice of the households about prevention of cutaneous leishmaniasis, Iran, Shiraz at 2016. *J. Hum. Environ. Health Promot.* **2**(3), 186–192 (2017). 10.29252/jhehp.2.3.186

[11] Sarkari, B., Qasem, A. & Shafaf, M. R. Knowledge, attitude, and practices related to cutaneous leishmaniasis in an endemic focus of cutaneous leishmaniasis, Southern Iran. *Asian Pac. J. Trop. Biomed.* **4**(7), 566–569 (2014). 25183278 10.12980/APJTB.4.2014C744 PMC4032832

[12] Vahabi, A. *et al.* First survey on knowledge, attitude and practice about cutaneous leishmaniasis among dwellers of Musian district, Dehloran County, Southwestern of Iran, 2011. *Life Sci J* **10**(12), 864–868 (2013).

[13] Hubley, J. Understanding behaviour: The key to successful health education. *Trop. Doct.* **18**(3), 134–138 (1988). 3406993 10.1177/004947558801800316

[14] Saghafipour, A. *et al.* The effectiveness of education based on BASNEF model on promoting preventive behavior of cutaneous leishmaniasis in students. *Int. J. Pediatr.* **5**(6), 5125–5136 (2017).

[15] Alizadeh, G., Shahnazi, H. & Hassanzadeh, A. Application of BASNEF model in students training regarding cutaneous leishmaniasis prevention behaviors: A school-based quasi experimental study. *BMC Infect. Dis.* **21**, 1–9 (2021). 34789186 10.1186/s12879-021-06874-2 PMC8596857

[16] Ajzen, I. The theory of planned behavior: Frequently asked questions. *Hum. Behav. Emerging Technol.* **2**(4), 314–324 (2020). 10.1002/hbe2.195

[17] Ghodsi, M., Joveiny, H., Rakhshani, M. H., Hoseini, Z. S. & Hashemifard, T. A M: Applying and comparing the standard health model and BASNEF model in predicting preventive behaviors of cutaneous leishmaniasis in students of Neyshabur city. *J. Neyshabur. Univ. Med. Sci.* **9**(2), 126–136 (2021).

[18] Nejadsadeghi, E. & Zubaidi, M. Cutaneous leishmaniasis preventive behaviors and the related factors among parents: Application of BASNEF model. *Int. J. Pediatr.* **10**(7), 16417–16427 (2022).

[19] Zeinali, M., Mohebali, M., Mahmoudi, M., Hassanpour, G. R. & Shirzadi, M. R. Study on Knowledge, attitude and practice of health workers of east Azerbaijan, Ilam and Khorasan Razavi provinces about leishmaniasis during 2015–2016: A comparative study before and after intervention. *Arch. Clin. Infect. Dis.* **14**(1), e64282 (2019).

[20] Ghodsi, M., Maheri, M., Joveini, H., Rakhshani, M. H. & Mehri, A. Designing and evaluating educational intervention to improve preventive behavior against cutaneous leishmaniasis in endemic areas in Iran. *Osong Publ. Health Res. Perspect.* **10**(4), 253 (2019). 10.24171/j.phrp.2019.10.4.09 PMC6711717 31497498

[21] Moussa, S. *et al.* Awareness and behavioral practice of cutaneous leishmaniasis among hail population, Kingdom of Saudi Arabia. *J. Microbiol. Exp.* **7**(2), 88–89 (2019).

[22] Heshmati, H., Charkazi, A., Hazavehei, S., Rahaei, Z. & Dehnadi, A. Factors related to cutaneous leishmaniasis preventive behaviors on the basis of BASNEF model in residents of endemic areas in Yazd Iran. *J. Res. Health Syst.* **7**(6), 926–934 (2011).

[23] Jafarpour, M., Aivazi, A., Jalali, A. & Ghazanfari, Z. Assessing preventive behaviors of leishmaniasis in Mehran county at 2014: Application of BASNEF model. *J. Ilam Univ. Med. Sci.* **25**(3), 23–31 (2017).

[24] Khosravani Poor, H. A., Ghavam, A. & Yazdan Panah, A. The impact of factors related to preventive behaviors of Cutaneous leishmaniasis among families of Kherameh 2015. *Pars J. Med. Sci.* **14**(1), 64–71 (2022).

[25] Coleman, M. T. & Pasternak, R. H. Effective strategies for behavior change. *Prim. Care Clin. Off. Pract.* **39**(2), 281–305 (2012). 10.1016/j.pop.2012.03.004 22608867

[26] Kabodi, S., Hazavehei, M. M., Rahimi, M. & Roshanaei, G. Application of BASNEF model in analyzing self-treatment behavior among type 2 diabetic patients in 2014. *J. Edu. Commun. Health* **2**(1), 38–49 (2015).

[27] Omidi, S., Farmanbar, R. & Mokhtarpour, S. The effect of educational intervention based on PRECEDE-PROCEED model on promoting traffic safety behaviors in primary schools students of Tabriz in 2014. *J. Edu. Commun. Health* **2**(4), 48–56 (2016). 10.21859/jech-02047

[28] Jafari, A. & Peyman, N. Application of theories/models of health education and promotion in health literacy research: A systematic review. *J. Health Lit.* **3**(2), 124–136 (2018).

[29] Alidosti, M., Heidari, Z., Shahnazi, H. & Zamani-Alavijeh, F. Behaviors and perceptions related to cutaneous leishmaniasis in endemic areas of the world: A review. *Acta Trop.* **223**, 106090 (2021). 34389332 10.1016/j.actatropica.2021.106090

