# Author Correction: Prediction of preventive behaviors of cutaneous leishmaniasis in the endemic areas of northwest Iran: an application of BASNEF model

**DOI:** 10.1038/s41598-024-66729-z

**Published:** 2024-07-19

**Authors:** Eslam Moradi-Asl, Arman Latifi, Mahsa Rashtbari, Abbas Abbasi-Ghahramanloo, Sara Rahimi

**Affiliations:** 1 Arthropod-Borne Diseases Research Center, Ardabil University of Medical Sciences, Ardabil, Iran; 2 Department of Public Health, School of Health, Ardabil University of Medical Sciences, Ardabil, Iran; 3 Department of Public Health, Research Center for Evidence-Based Health Management, Maragheh University of Medical Sciences, Maragheh, Iran; 4 Medicinal Plants Research Center, Maragheh University of Medical Sciences, Maragheh, Iran

Correction to: *Scientific Reports* 10.1038/s41598-024-64600-9, published online 15 June 2024

In the original version of this Article, Sara Rahimi was omitted as a corresponding author. Correspondence and requests for materials should also be addressed to rahimisaraa@gmail.com.

The original Article has been corrected.

